# Protein Kinase D regulates several aspects of development in *Drosophila melanogaster*

**DOI:** 10.1186/1471-213X-7-74

**Published:** 2007-06-25

**Authors:** Dieter Maier, Anja C Nagel, Helena Gloc, Angelika Hausser, Sabrina J Kugler, Irmgard Wech, Anette Preiss

**Affiliations:** 1 Universität Hohenheim, Institut für Genetik (240), Garbenstr. 30, 70599 Stuttgart, Germany; 2 Universität Stuttgart, Institut für Zellbiologie und Immunologie, Allmandring 31, 70569 Stuttgart, Germany; 3 present address: Universitätsklinikum Würzburg, Medizinische Klinik II, Josef-Schneider-Str. 2, 97080 Würzburg, Germany

## Abstract

**Background:**

Protein Kinase D (PKD) is an effector of diacylglycerol-regulated signaling pathways. Three isoforms are known in mammals that have been linked to diverse cellular functions including regulation of cell proliferation, differentiation, motility and secretory transport from the trans-Golgi network to the plasma membrane. In *Drosophila*, there is a single PKD orthologue, whose broad expression implicates a more general role in development.

**Results:**

We have employed tissue specific overexpression of various PKD variants as well as tissue specific RNAi, in order to investigate the function of the PKD gene in *Drosophila*. Apart from a wild type (WT), a kinase dead (kd) and constitutively active (SE) *Drosophila *PKD variant, we also analyzed two human isoforms hPKD2 and hPKD3 for their capacity to substitute PKD activity in the fly. Overexpression of either WT or kd-PKD variants affected primarily wing vein development. However, overexpression of SE-PKD and PKD RNAi was deleterious. We observed tissue loss, wing defects and degeneration of the retina. The latter phenotype conforms to a role of PKD in the regulation of cytoskeletal dynamics. Strongest phenotypes were larval to pupal lethality. RNAi induced phenotypes could be rescued by a concurrent overexpression of *Drosophila *wild type PKD or either human isoform hPKD2 and hPKD3.

**Conclusion:**

Our data confirm the hypothesis that *Drosophila *PKD is a multifunctional kinase involved in diverse processes such as regulation of the cytoskeleton, cell proliferation and death as well as differentiation of various fly tissues.

## Background

Protein kinases D (PKD) are serine/threonine-specific kinases that belong to the subfamily of Ca(2+)/Calmodulin kinases. They are effectors in diacylglycerol-regulated signaling pathways. In mammals, three highly related PKDs 1–3 (in human named also PKCμ, PKD2 and PKCν) are known [1]. PKD contains two domains, a regulatory domain and a catalytic kinase domain. The regulatory domain inhibits the kinase domain, until the enzyme is activated by phosphorylation of two serine residues located within the kinase domain. Three ways have been described to activate PKD1 [2]. As consequence of a mitotic signal, diacylglycerol is generated by phospholipase C stimulation, resulting in the activation of either novel protein kinase C, PKCε or PKCη, which in turn phosphorylate PKD1 [3,4]. Alternatively, PKD1 is activated by Gβγ that binds to the regulatory domain, thereby abrogating its inhibitory function [5]. Finally, in response to genotoxic stress, the kinase domain can be released by Caspase-mediated cleavage [6,7]. However, a PKD homologue in *C. elegans *DKF-1 (D-kinase factor 1) is directly activated by phorbol-esters independent of PKC [8]. PKDs are found within different subcellular compartments in agreement with their multiple biological roles in highly diverse cellular processes including cell proliferation and apoptosis, cell migration, cellular differentiation and notably, cargo specific secretory transport from the trans-Golgi network (TGN) to the plasma membrane [1,2]. The involvement of PKD in the regulation of fission of secretory vesicles from TGN was deduced primarily from overexpression experiments of a presumptive dominant negative PKD variant, which bears a single amino acid substitution in the ATP binding domain and therefore lacks kinase activity ('kinase dead') [9-11]. 'Kinase dead' PKD interferes with the fission of vesicles at the TGN owing to a tubularization of Golgi membranes [10]. The relevance of these observations is strengthened by the finding that one of the physiological substrates of PKD1 and PKD2 is phosphatidylinositol-4 kinase III beta (PI4KIIIβ), which is central to Golgi structure and function [9]. Apart from its role in secretory transport, transgenic mouse models reveal the importance of PKD for differentiation of T lymphocytes [12]. Moreover, mutants of the corresponding *C. elegans *kinase DKF-1 displayed body paralysis, whereas overexpression caused growth defects [8].

The *Drosophila *genome harbors a single PKD homologue. As expected for a multifunctional protein, PKD is broadly expressed during development. A fraction of the PKD protein localizes to the Golgi compartment in agreement with a proposed role in secretory transport [13]. Hence, *Drosophila *may serve as model system to investigate the *in vivo *function of PKD. To this end, we have analyzed the phenotypic consequences of overexpression of wild type and mutant PKD variants on the development of a number of tissues, the consequences of tissue specific RNA-interference and the capacity of human hPKD2 and hPKD3 to substitute for *Drosophila *PKD. Only slight defects primarily during wing vein development were observed upon overexpression of wild type (WT) or kd-variants, whereas overexpression of a constitutively active form is deleterious to fly development, as is PKD RNAi. Our data are in accordance with a role for PKD in the regulation of cytoskeletal dynamics, cell proliferation and death and hence, the differentiation of various tissues during fly development.

## Results

### PKD overexpression constructs

**Figure 1:**
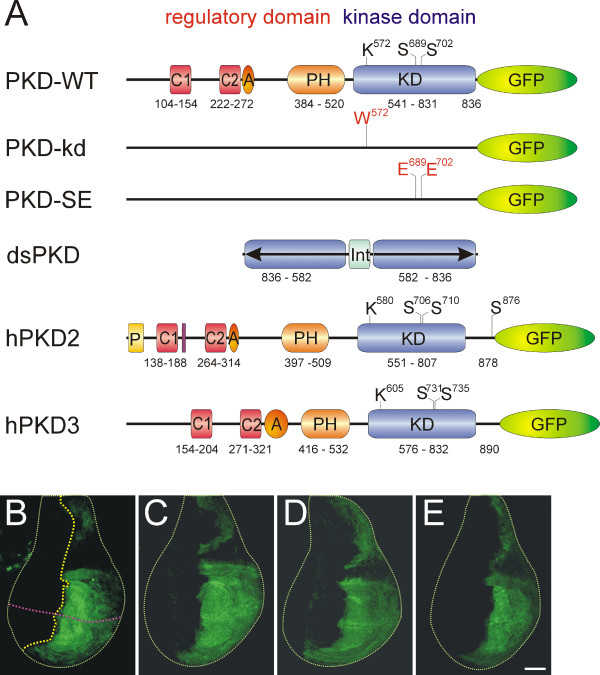
**PKD variants and activity**. A) Schematic representation of *Drosophila *wild type and mutant UAS-PKD variants, PKD-WT, PKD-kd, PKD-SE, dsPKD and human PKD variants, hPKD2 and hPKD3. Apart from dsPKD, all proteins were expressed as GFP fusion proteins. PKD contains a regulatory domain and a kinase domain. The characteristic features include: C1, C2, Zink-fingers 1 and 2; A, acidic domain; PH, pleckstrin homology domain; KD, kinase domain. In addition, hPKD2 contains a proline rich domain (P) at the N-terminal end and a serine rich region between the two zinc fingers (purple box). Two serine residues in the kinase domain (Ser_689_, Ser_702_) are thought to be phosphorylated upon activation of PKD. They were both mutated to glutamic acid in PKD-SE. hPKD2 contains a further possible auto-phosphorylation target within the C-terminus. The presumptive ATP binding site, lysine at the beginning of the kinase domain (K_572_), is highlighted. It was altered to tryptophane in PKD-kd. Numbers refer to codons; they are according to [1] for hPKDs and [13] for *Drosophila *PKD. B-E) Fidelity and level of expression for the different variants was analyzed *in vivo *by help of the GFP-tag. Different Gal4-driver lines were used to address tissue specificity of GFP-expression. The examples show specific GFP-expression in the posterior compartment of a wing imaginal disc (right half) as can be achieved by using the en-Gal4 driver line. For better visualization, discs are outlined with dotted lines. The antero-posterior (yellow line) and the dorso-ventral borders (purple line) are indicated in B). Shown are B) wild type PKD (en-Gal4/PKD-WT^10-1^), C) PKD-SE (en-Gal4/PKD-SE^112-6^), D) PKD-kd (en-Gal4/PKD-kd^102-1^) and E) hPKD3 (en-Gal4/hPKD3^X-1^). Scale bar, 100 μm.

Mammalian PKD is a multifunctional kinase regulating diverse processes including proliferation, apoptosis and secretory transport [1,2]. The lack of mutants hampers a direct analysis of PKD's role in *Drosophila*. In order to address the possible functions of PKD in the fly, we examined the consequences of overexpression of wild type, presumptive dominant negative and constitutively active PKD variants as well as of PKD depletion by RNA interference. Several UAS-constructs were generated (see Figure 1A) that allow tissue specific overexpression using the Gal4/UAS-system [14]. The PKD-WT construct expresses the PKD wild type protein fused to GFP [13]. To generate a 'kinase dead' (kd) PKD-construct, lysine K_572 _was replaced by tryptophan in order to destroy the presumptive ATP-binding domain. The resultant PKD protein was expected to be a dominant negative isoform according to earlier reports on respective changes in mammalian PKDs [9-11,15]. A constitutive active protein PKD-SE was generated by replacing two serine residues S_698 _and S_702 _with glutamate. These two serines are located within the activation loop and are presumably phosphorylated upon activation of PKD in analogy to respective observations on mammalian PKDs [13,16]. Likewise changes have been reported to generate constitutive active forms of human PKD as well [16,17]. Both variants, PKD-kd and PKD-SE were made as C-terminal fusion to GFP in order to facilitate their *in vivo *detection (Fig. 1A). The human isoforms hPKD2 and hPKD3 (i.e. PKD2 and PKCν respectively) [18,19] were likewise fused to GFP and cloned as UAS-overexpression constructs. They were chosen as a more diverse (hPKD2) and the most similar (hPKD3) in comparison with *Drosophila *PKD [13]. Finally, a large portion of the kinase domain (codons 582 – 836) [13] was cloned in inverted and direct orientation, split by a small intron into pUAST to generate dsPKD. This construct is expected to produce a double stranded RNA transcript [20], resulting in tissue specific RNA interference and hence, depletion of PKD activity. Figure 1A gives an overview of the different constructs. Several independent transgenic lines were generated from each construct and compared for their expressivity. PKD expression was monitored on Western blots and *in situ *in larval tissues making use of the GFP-tag (Fig. 1B–E, and data not shown) [13]. Finally, at least five strains of each construct were compared in overexpression experiments for their phenotypes; typical strains were used in subsequent experiments.

### Activity of PKD-kd and PKD-SE constructs in human cell culture

**Figure 2:**
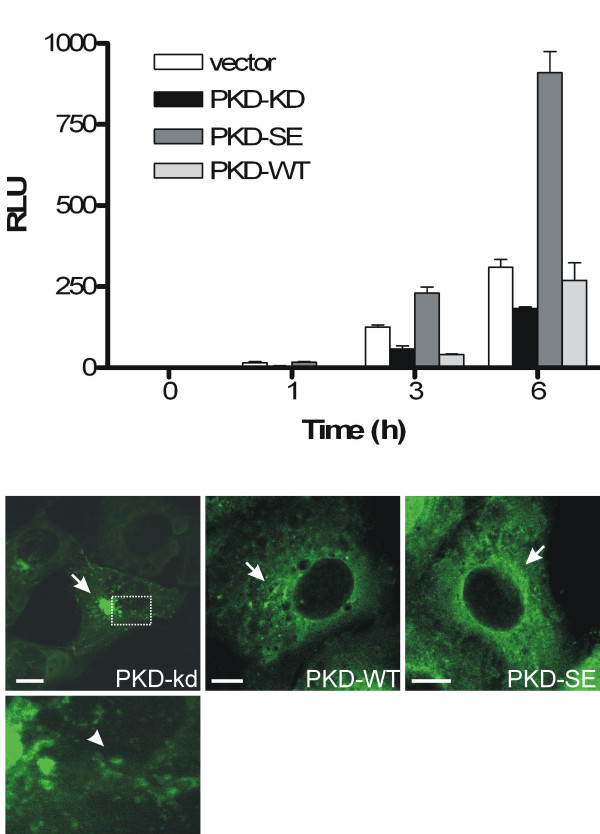
**Activity of PKD-kd and PKD-SE in cell culture**. A) HEK293T cells were cotransfected with an expression plasmid encoding Flag-ss-HRP or empty vector, and PKD-kd, PKD-SE, or PKD-WT respectively. 24 h post-transfection, cells were washed and fresh medium was added. The supernatant was analyzed for peroxidase activity after 0, 1, 3 and 6 h by chemiluminescence. Values correspond to the mean of triplicate samples and error bars represent standard deviation. RLU, relative light units. B) COS7 cells were transiently transfected with expression plasmids encoding GFP-tagged PKD-kd, PKD-WT and PKD-SE. Cells were fixed 24 h post transfection and analyzed. The images shown are stacks of several confocal sections. Arrow points to TGN. Enlargement (boxed) of tubules (arrowhead) induced by PKD-kd overexpression is shown below the figure. Scale bar, 10 μm.

Human PKD has been reported to regulate secretory transport from the TGN to the plasma membrane [10]. To investigate whether the PKD-kd and -SE variants are interfering with secretory transport, we made use of a plasmid encoding horseradish peroxidase (HRP) fused to a signal sequence. The fusion protein ss-HRP can be used as a reporter for constitutive protein secretion [21]. We transfected HEK293 cells with plasmids encoding for ss-HRP and PKD-WT, -kd or -SE and measured the secretion of ss-HRP over time. In control cells, secretion of ss-HRP could be detected within 1 hour and increased over time (Fig. 2A). Co-expression of PKD-kd inhibited the secretion of ss-HRP into the supernatant, whereas co-expression of PKD-SE increased the amount of secreted HRP compared to the control. Expression of PKD-WT had no effect on secretion of ss-HRP in this system. It has been demonstrated, that expression of a kinase dead PKD variant leads to tubularization of the TGN [10]. We therefore investigated, whether expression of *Drosophila *PKD-kd displays similar effects. Unfortunately, changes in Golgi-morphology are difficult to detect in *Drosophila *cells due to their small size [21,22], which hampers an analysis in this system. Therefore we transfected COS7 cells with plasmids encoding *Drosophila *PKD-WT, -kd and -SE proteins fused to GFP. PKD-WT and PKD-SE displayed a similar localization; both proteins were predominantly localized in the cytoplasm with a small portion of the protein enriched at a perinuclear structure, which presents the Golgi compartment ([13] and Fig. 2B). In contrast, PKD-kd was strongly enriched at the Golgi compartment and induced the formation of tubules. This is in accordance with the phenotype induced by expression of mammalian dominant negative PKD-kd variants [10].

### Overexpression of PKD interferes with wing development

**Figure 3:**
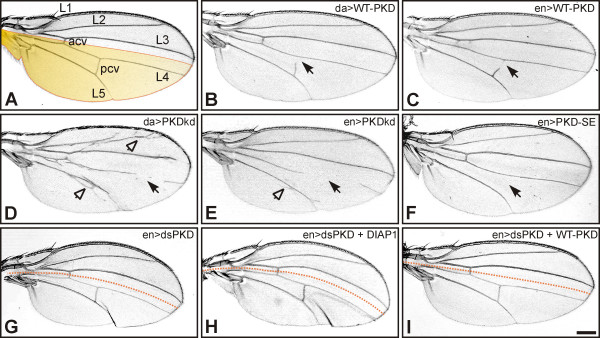
**Defects in wing development upon PKD overexpression**. A) Wild type wing with five longitudinal (L1 – L5) and anterior and posterior cross veins (acv, pcv). The posterior compartment is highlighted in yellow. B) Ubiquitous PKD-WT overexpression affects the posterior cross vein (arrow). C) A similar defect is observed upon overexpression of PKD-WT just in the posterior compartment (arrow). D) Overexpression of PKD-kd caused loss of veins (arrow) and induced ectopic veinlets (arrowhead). E) In contrast, overexpression of PKD-kd within the posterior compartment only resulted primarily in loss of veins (arrow) and only few ectopic veinlets (arrowhead). F) Overexpression of PKD-SE within the posterior compartment prevented cross vein formation (arrow). G) Depletion of PKD within the posterior wing compartment by RNAi caused loss of wing tissue, resulting in a conspicuous bent of the wing when compared to wild type. It did not affect wing venation; however, the texture of the wing was altered. A dotted line marks the antero-posterior compartment border. H) Tissue loss was not influenced by concomitant overexpression of DIAP1, albeit the total size of the wing was increased (compare with G). I) In contrast, a complete rescue was observed by concurrent overexpression of PKD-WT. Genotypes are: A) da-Gal4/+, UAS-GFP/+; B) da-Gal4/UAS-PKD-WT^10-1^; C) en-Gal4/UAS-PKD-WT^10-1^; D) da-Gal4/UAS-PKD-kd^102-1^; E) en-Gal4/UAS-PKD-kd^102-1^; F) en-Gal4/+, UAS-PKD-SE^112-6 ^/+; G) en-Gal4/+, UAS-dsPKD^124-4 ^/+; H) UAS-DIAP1/+, en-Gal4/+, UAS-dsPKD^124-4 ^/+; I) en-Gal4/UAS-PKD-WT^10-1^, UAS-dsPKD^124-4 ^/+. Scale bar, 200 μm.

In a first set of experiments, the consequences of overexpression of PKD variants on adult flies were monitored. The PKD variants were ubiquitously overexpressed using the da-Gal4 driver line and within the posterior wing compartment using the en-Gal4 driver line, respectively. Induction of either PKD-WT or PKD-kd variants interfered with formation of wing veins (Figure 3B–E). The posterior cross vein was frequently affected and incompletely developed. In addition to massive vein loss, PKD-kd induced ectopic veins, mostly abutting the second and less often the fifth longitudial vein, when overexpressed uniformly during larval development (Figure 3D,E).

Ubiquitous overexpression of PKD-SE and dsPKD disrupted fly development. Offspring from a cross of da-Gal4 with UAS-PKD-SE all died at first larval instar larva at 18°C and at 25°C. Most da > dsPKD animals died as prepupae at 25°C and only few started pupation (13 of 207); at 18°C they developed into pharate adults with few attempting to hatch (5 of 164). Induced within the posterior compartment only, PKD-SE was fully lethal at late pupal stage at 25°C and semilethal at 18°C (32 of 224 animals hatched). Many of the eclosed animals were defective in wing inflation; unfolded wings typically showed a complete lack of cross veins (Fig. 3F). Hence, cross vein formation requires a tight regulation of PKD activity. The ectopic veins that were caused by overexpression of PKD-kd may reflect dominant negative effects.

**Figure 4:**
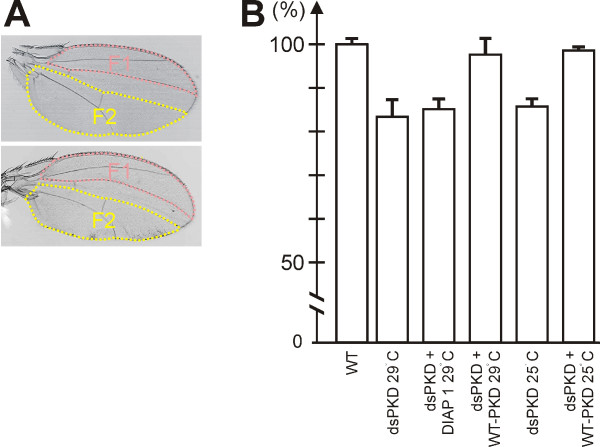
**Rescue of wing defects**. A) Calculation of RNAi-effects within the posterior compartment. Two examples are shown, a control wing (upper) and a mutant wing (lower). Anterior (F1, pink) and posterior (F2, yellow) compartment area (in pixels) was measured as depicted. The area between L3 and L4 was ignored since the antero-posterior boundary cannot be exactly determined. The F2/F1 ratio was calculated for each wing independently to avoid influence of natural variation on wing size e.g. due to nutritional conditions. B) PKD-RNAi was induced in the posterior compartment at 29°C or 25°C as indicated; concurrently, PKD-WT10-2 or DIAP1 were overexpressed. A minimum of five wings of each genotype was measured as depicted in a) and the mean value of F2/F1 was determined. The wild type ratio was taken as 100% and the others calculated as percentage of wild type. Error bars represent standard deviation.

Overexpression of dsPKD in the posterior compartment effected slightly undulated wings that were somewhat shorter than the control and bent towards the posterior (Figure 3G). Wing curvature was a consequence of a smaller posterior compartment (compare Fig. 3A and 3G, Fig. 4). The overall size of the posterior compartment was decreased by about 20% compared to a wild type wing (Fig. 4). However, cell number values (cells per area) were unchanged compared to wild type or to the anterior compartment suggesting that cell size remained constant in contrast to overall cell number. Cell numbers could be due to lowered proliferation and/or induction of apoptosis. Tissue loss was rescued by PKD-WT indicating that it was indeed caused by PKD depletion (Fig. 3I and Fig. 4). We addressed the involvement of apoptosis by concomitant overexpression of *Drosophila *inhibitor of apoptosis 1 (DIAP1). In none of the experiments performed we observed a full rescue (Fig. 3G,H; Fig. 4 and data not shown). However, we noted a slight improvement: although the wing bent remained unchanged despite DIAP1 overexpression, the overall size of the wing was increased (Fig. 3H). Comparing the anterior compartments only, en-Gal > dsPKD was 88.6% (± 6.4% SD) of wild type size and increased to 96.1% (± 4.5% SD) by concurrent overexpression of DIAP1.

### PKD specific RNA interference during larval development results in loss of tissue

**Figure 5:**
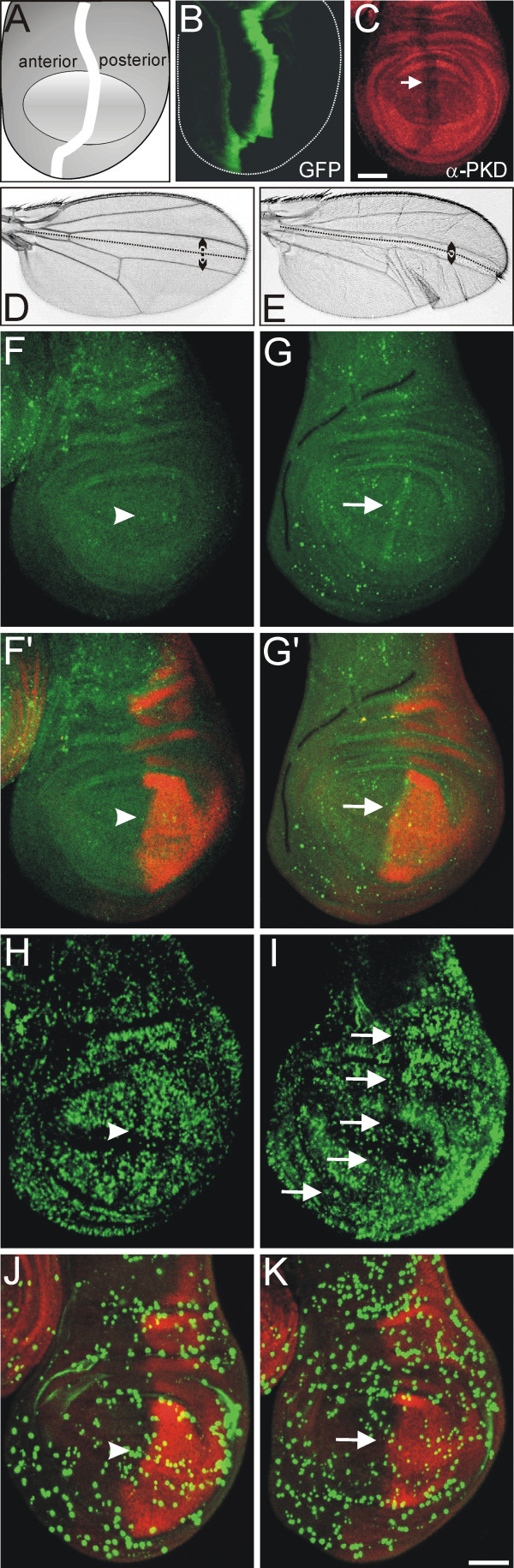
**Loss of tissue upon PKD RNAi**. A) Schematic drawing of a wing imaginal disc, highlighting the antero-posterior (a/p) boundary. Oval represents presumptive wing blade. B) Expression domain of ptc-Gal4 along the a/p boundary, visualized by overexpression of GFP (green). C) RNA interference was induced by overexpression of UAS-dsPKD^114-5 ^with ptc-Gal4 and caused detectable reduction of PKD protein accumulation (red; arrow). D) Control wing; the antero-posterior boundary is marked by a dotted line; the distance between third and fourth longitudinal veins is highlighted by a double-headed arrow. E) PKD RNAi along the antero-posterior boundary results in tissue loss (compare double-headed arrow with control in D) and in a folded wing with uneven texture. F – J) Analysis of cell proliferation and apoptosis in wing discs of ptc > dsPKD larvae (G, G', I, K) versus control discs (F, F', H, J). Antibody staining against engrailed (red; F', G', J, K) highlights the border of the posterior compartment. Scale bar, 100 μm. F, F') Control wing disc stained for activated Caspase 3 (green) to label apoptotic cells and for engrailed (red) to mark the border of the posterior compartment (arrowhead). G, G') A likewise stained wing disc of ptc > dsPKD larvae reveals an enrichment of activated Caspase 3 along the antero-posterior boundary (arrow). H) BrdU-incorporation (green) was visualized to reveal replicating cells in a control disc. Note the zone of non-proliferation along the dorso-ventral boundary as well as a slight decrease of S-phase cells along the antero-posterior boundary (arrowhead). I) In contrast to the control, wing discs of ptc > dsPKD larvae show a decrease of BrdU incorporation all along the ptc-stripe (arrows) indicating a downregulation of cell division. J) Control wing disc stained for phospho-Histon H3 (green) to label mitotic nuclei; the posterior compartment is highlighted in red (arrowhead). K) A likewise stained wing disc of a ptc > dsPKD larva shows no overt difference in mitotic cells compared to control neither with regard to total number nor along the antero-posterior border (arrow). Genotypes are: B) ptc-Gal4/+, UAS-GFP/+; C, E, G, G', I, K) ptc-Gal4/+, UAS-dsPKD^6-4 ^/UAS-dsPKD^6-4^; D, F, F', H, J) ptc-Gal4/+.

The smaller posterior wing compartment caused by overexpression of dsPKD conforms to an involvement of PKD in the regulation of cell proliferation or cell death in *Drosophila*. In order to corroborate this finding we investigated further driver lines for similar phenotypes. When two copies of dsPKD were overexpressed along the antero-posterior border in the wing anlagen (Fig. 5A–C), the affected tissue, which is normally located between the third and the fourth longitudinal vein, was partially lost in adult wings (Fig. 5D,E). In addition, the wings were undulated and uneven in texture. In this setting, we could also show a reduction of PKD protein expression indicating that overexpression of the RNAi construct caused indeed a hypomorphic situation (Fig. 5A–C). However, larval wing imaginal discs appeared fairly normal in size and the strip of cells, where RNAi was induced, appeared as wide as in the control suggesting that the developmental defects occurred primarily during pupal morphogenesis (compare Fig. 5B,C). We noted enrichment of activated Caspase 3 along the antero-posterior boundary indicative of apoptosis (Fig. 5F–G). At the same time, we observed a slight decrease in BrdU incorporation, although no conspicuous reduction in the number of mitotic cells was observed (Fig. 5H–K). Regarding all this, we conclude that both, impaired proliferation as well as increased apoptosis was involved in generating tissue loss in the adult wing. The partial rescue may result from insufficient DIAP1 doses or from additional stronger defects in cell proliferation primarily during metamorphosis, contributing to the tissue loss phenotype. The reduction of tissue size was not restricted to the wing: we observed a dramatic reduction of the adult eye when dsPKD was overexpressed in the eye anlagen using the ey-Gal4 driver line, too (not shown).

### Human PKD can rescue loss of *Drosophila *PKD activity

Overexpression of *Drosophila *dsPKD along the antero-posterior border in the wing imaginal discs caused a fully penetrant wrinkled wing phenotype that was amenable to further genetic studies (Fig. 5E). We used this phenotype to address the question whether human hPKD2 or hPKD3 was able to replace *Drosophila *PKD function. This was indeed the case. Like *Drosophila *PKD-WT, both human PKDs rescued the ptc > dsPKD wing phenotype to a similar degree (Table 1). Overexpression of either construct did not effect an apparent wing phenotype on its own. Human PKD is thought to act downstream of Phospholipase C and novel Protein Kinase C (nPKC) [1]. Moreover, hPKD1 and hPKD2 have been recently shown to target PI4KIIIβ [9]. However, mutants or overexpression constructs of the respective fly homologues are not yet available. In addition, we asked whether concurrent overexpression of the PKC-inhibitor PKCi increased or decreased the PKD-RNAi defects, neither was the case (Table 1). We conclude that PKC is not closely related to PKD signaling at least not during wing formation in *Drosophila*.

**Table 1:** Human PKD can replace *Drosophila *PKD activity

**UAS-Line**	**n**	**0/1**	**2**	**3**
GFP-lacZ	19	0%	0%	100%
PKD-WT^10-2^	44	77,2%	4,6%	18,2%
PKD-SE^139-2^	35	94,3%	2,9%	2,8%
hPKD2^II-3^	22	63,6%	36,4%	0%
hPKD2^X-2^	16	89,0%	8,3%	2,7%
hPKD3^V-1^	28	79,6%	14,3%	6,1%
hPKD3^X-1^	21	86,5%	8,1%	5,4%
PKCi	23	0%	0%	100%

The given UAS-line was overexpressed along the antero-posterior border with help of the ptc-Gal4 driver line together with UAS-dsPKD^6-4^. As control, ptc > dsPKD^6-4 ^was crossed to UAS-GFP-lacZ and wings used for comparison. Wings of at least ten flies were mounted and examined at high magnification. n, number of wings scored. 0/1, wild type looking wing; 2, weak phenotype; 3, phenotype indistinguishable from control. Numbers represent percentage of wings with given phenotype.

### Manipulation of PKD activity results in degeneration of the adult retina

**Figure 6:**
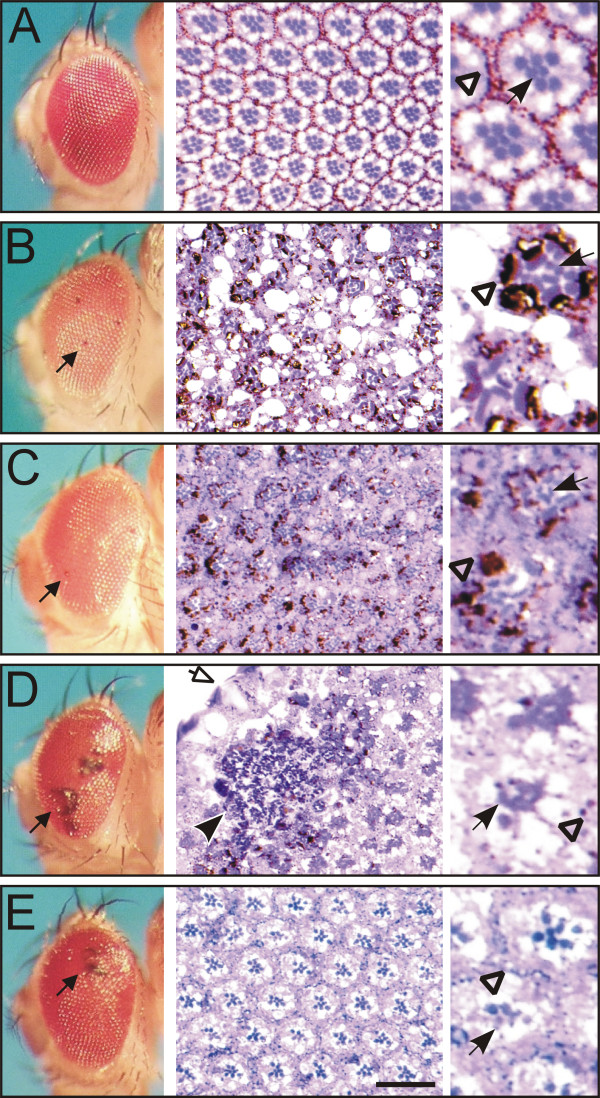
**Manipulation of PKD activity causes retina degeneration. **A) Eyes of control flies have externally a smooth and regular appearance (left column). Tangential sections reveal the underlying, semi-crystalline architecture of the facets (center column). Seven photoreceptor cells can be distinguished by the centrally located rhabdomeres (arrow) that contain the light sensitive rhodopsin. Each facet is insulated by surrounding pigment cells that contain pigment granules (arrowhead). See enlargement in the right column. B) Overexpression of one or several copies PKD-SE^139-2 ^caused single black dots that have the size of a single facet (small arrow). Tangential sections reveal degeneration of the retina. The borders between ommatidia become blurred by many holes; also, fusion was observed. The pigment granules seem to clump and no longer encircle the rhabdomeres (arrowhead). The rhabdomeres are swollen and elongated; some are fragmented (arrow). Some rhabdomeres are absent and others degenerated. C) The defects remain largely unaltered when DIAP1 is overexpressed simultaneously, apart from a good rescue of the holes. D) Overexpression of dsPKD causes large black patches with a glossy surface (small arrow). Sections reveal massive amounts of cocci within the black patch (black arrowhead) underlying thinner lens (open arrow). Rhabdomeres are collapsed and degenerated (arrow); sporadic pigment granules can be seen (arrowhead). E) Concurrent overexpression of DIAP1 has little influence on the superficial appearance (arrow). However, the retina is somewhat more regular, which is reflected by the pigment granules that partly encircle the ommatidia (arrowhead). Moreover, rhabdomeres are well separated but many of them are degenerated (arrow). Genotypes are: A) wild type; B) gmr-Gal4/PKD-SE^139-2^; C) UAS-DIAP1/+, gmr-Gal4/PKD-SE^139-2^; D) gmr-Gal4/+, UAS-dsPKD^114-5 ^/+; D) UAS-DIAP1/+, gmr-Gal4/+, UAS-dsPKD^114-5 ^/+. Scale bar, 20 μm.

Human PKD is known to be involved in multiple processes, most notably in secretory transport from the trans-Golgi network to the plasma membrane [10,11]. Recently, we have found that the expression patterns of *Drosophila *PKD are compatible with a role in secretory transport [13], which we aimed to address experimentally. Therefore, we investigated the consequences of a manipulation of PKD activity on the adult retina. During late pupal development, rhodopsin and other phototransduction proteins are transported from the Golgi into rhabdomeres, the light sensitive membranes of the photoreceptor cells [23]. A block in maturation or trafficking of rhodopsin results in a degeneration of the rhabdomeres and subsequently the retina in a light dependent manner over time [23-25]. This can be shown in sections of adult eyes. We used the gmr-Gal4 driver line to induce the expression of the PKD proteins behind the morphogenetic furrow within cells that enter neuronal differentiation. No effects on the outer eye were observed upon induction of *Drosophila *PKD WT, human hPKD2 and hPKD3 or of the PKD-kd variant. However, we noted small black dots that were restricted to single ommatidia when PKD-SE was overexpressed (Fig. 6A,B). Induction of dsPKD in the gmr-pattern affected the outer eye more dramatically: about a third of the flies had one or more glossy shining patches in their eyes that turned dark over time (Tables 2, 3). Patches appeared necrotic and irregular (Fig. 6D). These outer eye phenotypes were largely insensitive to inhibition of apoptosis (Fig. 6B–E; Table 2).

**Table 2:** Rescue of external eye phenotypes by *Drosophila *inhibitor of apoptosis DIAP1

genotype^a^	phenotype^b^	total number^c^
1) PKD-SE	31,9%	238
2) DIAP1 + PKD-SE	30,4%	194
3) dsPKD	19,7%	259
4) DIAP1 + dsPKD	19%	252

(a) Genotypes: 1) PKD-SE: gmr-Gal4/PKD-SE139-2; 2) DIAP1+ PKD-SE: UAS-DIAP1/+; gmr-Gal4/PKD-SE139-2; 3) dsPKD: gmr-Gal4/+; dsPKD114-5/+; 4) DIAP1 + dsPKD: UAS-DIAP1/+; gmr-Gal4/+; dsPKD114-5/+. (b) For PKD-SE, eyes with black dots were counted and for gmr > PKD-SE, glossy and/or black patches, respectively. (c) Total number represents the number of flies of a given genotype scored.

**Table 3:** Age dependency of external eye phenotypes

	**gmr > PKD-SE**^a^		**gmr > dsPKD**^b^			
**days AE**^c^	N	1–4 spots^d^	n	1–2 spots^e^	≥ 3 spots	total

**0–3 d**	117	29,1%	170	3%	0%	3%
**12–15 d**	101	22,8%	248	19,3%	7,7%	27%
**21 d**	Nd	-	137	5,8%	29,2%	35%

Genotypes are gmr-Gal4/UAS-PKD-SE139-2 (a) and gmr-Gal4/+; dsPKD114-5/+ (b). Flies were analyzed at given times after eclosion (days AE) (c). Crosses were set up in parallel and performed at 25°C. External phenotypes scored for gmr > PKD-SE were dark spots (D) and for gmr > dsPKD fully black patches (e). nd, not determined.

Sections of the adult eyes revealed retina defects for both genotypes (see below). We noted thinner lenses in some ommatidia of gmr > dsPKD flies (Fig. 6B). The large black patches in older flies were filled with cocci (Fig. 6D). Bacterial infection can be an explanation for the darkening and increase in size of these patches with age and their resistance to rescue by DIAP1 (Fig. 6D,E; Tables 2, 3). The observed lesions in the eye lens may damage the physical barrier of the cuticle, thereby allowing bacterial entry more easily. Accordingly, other obvious infections were not prevalent in dsPKD flies compared to their untreated siblings. Alternatively, PKD could be involved in fly immune response. However, no rescue was seen with UAS-defensin, which has been shown to specifically combat gram^+^bacteria [26].

Retina defects were analyzed in sections. PKD-SE overexpression induced striking elongation and fragmentation of rhabdomeres (Fig. 6B), whereas PKD-RNAi resulted in a collapse and disappearance of rhabdomeres (Fig. 6D). Conspicuous holes were detected between the ommatidia in both cases (Fig. 6B,D). Since the overall ommatidial architecture appeared quite normal at low resolution, we conclude that the retina degenerated during metamorphosis after being established normally during larval and early pupal development. Accordingly, photoreceptor cells appeared normal when visualized with anti-Elav antisera in third instar larval eye discs of gmr > PKD-SE animals (data not shown). Sections revealed a slight improvement by concomitant overexpression of DIAP1; most notably the holes disappeared (Fig. 6C,E). However, DIAP1 had little effect on the morphology of the rhabdomeres. Neither could it halt the fragmentation caused by overexpression of PKD-SE nor the disintegrating resulting from PKD-RNAi (Fig. 6C,E). Altogether, these data suggest that the degeneration of the adult retina is partly caused by apoptosis, yet other mechanisms must contribute as well. This was unexpected since retinal degeneration caused for example by rhodopsin mutations can be prevented by blocking apoptosis [27].

**Figure 7:**
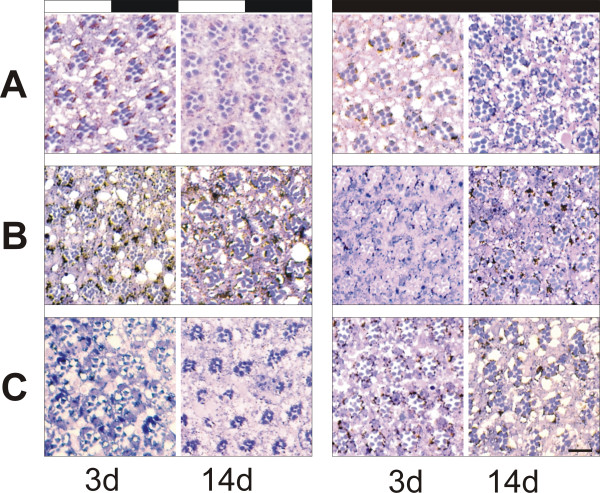
**Dependence of retinal degeneration on light and age. **Flies were raised in twelve-hour day-night cycle (left column; labelled with black and white bar) or in complete darkness (right column; black bar) and aged for several days at 25°C. Time points shown: 3d, 0–3 days after hatching; 14d, 10–14 days after hatching. Enlargements of each 4 × 4 ommatida are shown to highlight the defects. A) Eyes of control flies: Note the regular architecture of ommatidia, which is independent of light and age. B) PKD-SE was overexpressed behind the morphogenetic furrow. In the light (left), rhabdomeres are elongated and sometimes fragmented or completely absent. In the dark (right) these phenotypes are less extreme but still detectable. They are largely independent of age. C) dsPKD was likewise induced in a light-dark cycle (left) and in the dark (right). Note degeneration of rhabdomeres in the light reared, 14 days old flies. The degeneration is less severe in younger flies or those kept in the dark. Genotypes are: A) gmr-Gal4/+; B) gmr-Gal4/PKD-SE^139-2^; C) gmr-Gal4/+, UAS-dsPKD^114-5 ^/+. Scale bar, 10 μm.

We next asked, whether PKD-mediated retinal degeneration was light and age dependent, which is typical for defects in rhodopsin maturation or trafficking [23,24]. We set up parallel experiments, in which we overexpressed PKD-SE and dsPKD in complete darkness and in a normal day-night light cycle, and examined the subsequent retinal degeneration at the day of eclosion and 3, 6, 10 and 14 days later, respectively (Fig. 7, and data not shown). We noticed that the effects of PKD-SE were largely independent of age, however, were more severe in the light than in the dark. Whereas the rhabdomeres of the light-reared flies were elongated and often fragmented, those of the dark reared flies appeared swollen and irregular. Fragmentation was less frequent. However, retina degeneration occurred also in the dark albeit it was less severe (Fig. 7B). In contrast, the defects caused by PKD-RNAi were both dependent on light and age since the strongest phenotypes were observed in the older flies that were reared in a day-night cycle. In these flies, many rhabdomeres had completely disappeared. In contrast, younger flies or flies reared in the dark showed much milder signs of deterioration (Fig. 7C). Hence, PKD is important for the formation and the maintenance of rhabdomeres where it seems to be continuously required in light sensitive processes.

## Discussion

### *Drosophila *PKD affects wing development

Overexpression of both wild type and the kd-PKD isoforms interfered in particular with wing vein development. Wing veins are established in several phases during larval and pupal development and require the activity of a number of signaling pathways [28-30]. One example is the Notch signaling pathway. Overactivation of this pathway results in loss of veins indistinguishable of phenotypes observed for example in en > PKD-kd (Fig. 3E) [31,32]. However, ectopic expression of proneural proteins can induce ectopic veinlets similar to what is seen in the da > PKD-kd wings [31]. Notch activity reduces those veinlets and hence, acts in the opposite direction. Therefore, PKD might positively regulate Notch signaling, whereas the dominant negative activity of PKD-kd might inhibit it. Similar phenotypes can also be a consequence of disturbance of EGFR-signaling. For example, loss of veins is seen in *veinlet (ve) *mutants whereas overexpression of *ve *induces ectopic veins [31,33]. The Ve protein is a positive regulator of EGFR-signaling and is required for the release of the respective ligands [34]. Hence, PKD might be involved in the negative regulation of EGFR-signaling. Finally, the dpp-pathway plays an important role in the positioning and consolidation of veins and notably, of cross veins [28-30,35-37]. Interestingly, vein phenotypes caused by an overactivation of the dpp-pathway are indistinguishable from those caused by the overexpression of PKD-kd [29]. Any of these pathways or all of them may be influenced by PKD, which can be now addressed in more detail. As cross veins are most sensitive towards overexpression of PKD, the dpp-pathway seems to be the most promising candidate. The altered wing texture caused by overexpression of dsPKD appears to be non-autonomous and may reflect defects in the secretion of extracellular matrix or wing cuticle. Shortly after eclosion, wing epithelial cells delaminate and migrate into the thorax. Extra-cellular matrix components are produced presumably by these migrating cells that bond ventral and dorsal wing surfaces once the cells have disappeared [38]. Disturbance of this process interferes with wing unfolding and bonding [39]. The resultant phenotypes are reminiscent of defects seen upon overexpression of either PKD-SE (wing unfolding) or dsPKD (flaccid wings) and in agreement with a role for PKD in these processes.

### Human PKD can replace *Drosophila *PKD activity

Mammals contain three highly related PKD isoforms that, based on their similarity, may be functionally redundant. However, they are differentially expressed suggesting tissue specific activities [40]. The human isoforms, hPKD2 and hPKD3 rescued the wing phenotype caused by RNAi with *Drosophila *PKD (Table 1). *Drosophila *and human PKDs show an overall degree of similarity of about 65%, albeit the kinase domain is well-conserved [13]. The latter raises the possibility of an interference between dsPKD and hPKD mRNA. Stretches of identical sequences are rather short, however, may suffice for interference notably with hPKD2 and less likely with hPKD3 (see Materials and Methods) [41]. The similarity in rescue of either construct is in agreement with a replacement of *Drosophila *PKD function rather than a titration of the generated siRNAs by hPKD mRNA. Strikingly, we observed that both hPKD proteins were predominantly cytosolic in *Drosophila *tissues, just like the *Drosophila *PKD protein (Fig. 1B–E). In contrast to *Drosophila *PKD, hPKDs are nuclear and cytosolic in mammalian cells [2,13]. Mammalian cells use specific mechanisms to regulate nuclear import of PKD that may not operate in the fly [42]. Nuclear transport of PKD requires PKC and responds to G protein coupled receptor (GPCR) activation [43]. In this context, it is noteworthy that the *C. elegans *homologue DKF-1 is stimulated directly by phorbol-esters independent of PKC [8]. It is conceivable that *Drosophila *PKD is activated in a similar way.

### *Drosophila *PKD is required for the formation and maintenance of rhabdomeres

Both gain and loss of function of PKD as realized by the overexpression of the presumptive constitutively active PKD-SE isoform and by dsPKD-constructs, respectively, caused a degeneration of the adult retina. Apparently, the fly's retina is highly sensitive towards PKD doses. The phenotypes were quite distinct suggesting that they were not just a consequence of interference with the trafficking of rhodopsin. In this case, we would have expected a degeneration of rhabdomeres in a light and age dependent manner that, in addition, should be amenable to rescue by inhibition of apoptosis [23,24,27]. In contrast, the defects caused by the overexpression of the two PKD constructs were only slightly ameliorated by DIAP1. We cannot exclude, however, that the dose of DIAP1 was insufficient for a full rescue. Overall, the retinal degeneration seemed not to be caused primarily by interference with rhodopsin trafficking. Whereas PKD downregulation resembled defects in rhodopsin, the overactivation of PKD caused the elongation and sometimes fragmentation of the rhabdomeres. This phenotype is more reminiscent of mutations in *bifocal (bif) *and *Amphiphysin (Amph) *[44,45]. *Bif *encodes a presumptive cytoskeletal regulator and associates with actin filaments. Amph is localized to actin-rich membrane domains and is involved in their structural organization. Together, these two genes are required to organize a localized actin cytoskeleton and eventually form the microvilli stacks that build up the rhabdomeres [44,45]. Based on the similarity of the phenotypes, we propose that manipulation of PKD somehow affects the cytoskeleton and thereby the formation and maintenance of rhabdomeres rather than being involved in rhodopsin trafficking. In fact, rhodopsin itself plays an essential structural role in rhabdomere morphogenesis that involves F-actin and the unconventional myosin ninaC [25]. Accordingly, mutations affecting either rhodopsin, ninaC or components involved in establishment of the microtubule network, i.e. F-actin capping proteins, cause a likewise retinal degeneration [24,25,46]. An involvement of PKD in cytoskeletal dynamics would attribute to both, the gain and the loss of function phenotypes. Interestingly, mammalian PKD1 is associated with the F-actin binding proteins cortactin and paxillin in invasive breast cancer cells where it may regulate cell adhesion and motility [47]. In the context of photoreceptor axon guidance, Bif is phosphorylated and thereby regulated by the Ser/Thr kinase misshapen [48]. It is tempting to speculate that PKD might likewise regulate Bif, thereby implementing its influence on the actin cytoskeleton. Further investigations on the relationship of PKD and Bif in *Drosophila *may help to elucidate the role of human PKDs in the regulation of the cytoskeleton and metastasis.

## Conclusion

Our overexpression experiments reveal an involvement of *Drosophila *PKD in many aspects of development. The effects on vein formation argue for a regulatory input of PKD on one or several signaling pathways, for example dpp-, Notch- or EGFR-pathways. Tissue loss for example in the wing caused by PKD RNAi suggests a role in proliferation and regulation of apoptosis, which is corroborated by respective antibody stainings. A striking degeneration of the adult retina was observed upon downregulation of PKD by RNAi or by overexpression of the presumptive constitutively active PKD-SE isoform. In both cases the formation and maintenance of the rhabdomeres was affected. The observed phenotypes conform to a role of PKD in the regulation of actin dynamics in agreement with similar findings in mammalian cells. Although the eye phenotypes are most likely not a consequence of a disturbed trafficking of rhodopsin, PKD might be involved in the transport of other basolateral cargo for example in extracellular matrix formation of the adult wing. Despite a considerable divergence, human hPKD2 and hPKD3 could largely restore *Drosophila *PKD RNAi-phenotypes. In summary, our studies are in accordance with a role of PKD in the regulation of cell proliferation and death and hence, the differentiation of various tissues during *Drosophila *development. Most interestingly, the potential involvement of PKD in the regulation of cytoskeletal dynamics may help to unravel the role of human PKDs in cell motility and metastasis.

## Methods

### Fly strains and crosses

Flies were obtained from the Bloomington stock center, if not mentioned otherwise; information on strains can be found in the flybase. Tissue specific overexpression or RNAi induction was achieved with the Gal4/UAS system [14,20] using *da-Gal*, *en2.4-Gal4*^e16*E*^*, ey-Gal4 *(gift from U. Walldorf), *gmr-Gal4*, and *ptc*^559^*-Gal4 *as driver lines. Crosses were performed at 18°C, 25°C or 29°C. The following UAS-lines were used: UAS-PKD-WT [13], UAS-PKD-kd, UAS-PKD-SE, UAS-hPKD2, UAS-hPKD3, UAS-dsPKD (see below), UAS-DIAP1 (gift from A. Müller), UAS-defensin (gift from B. Lemaitre), UAS-PKCi.B^4*A *^and UAS-GFP.

### Generation of overexpression constructs and transgenic lines

PKD overexpression constructs fused to GFP were generated as outlined before [13]. Briefly, PKD cDNA was mutated to PKD-kd using the primer pair KWUP 5'-GAG GTG GCC ATC TGG GTG ATC GAC AAA -3' and KWLO 5'- TTT GTC GAT CAC CCA GAT GGC CAC CTC – 3' and to PKD-SE using the primer pair SEUP 5'- ATC GGC GAG AAG GAG TTC CGG CGC GAG GTG GTT GGC ACT -3' and SELO 5'-AGT GCC AAC CAC CTC GCG CCG GAA CTC CTT CTC GCC GAT -3' applying the QuickChange^® ^II XL site-directed mutagenesis kit (Stratagene). Mutant cDNA was shuttled into pEGFP-N1 vector (Clontech) and pUAST vector [14] as described for PKD-WT [13]. pUAST-hPKD2 and pUAST-hPKD3 were generated from plasmids pEGFP-N1-PKD2 and pEGFP-N1-PKD3 [9]; cDNA encoding hPKD2 was shuttled as *EcoR *I/*Not *I fragment into likewise digested pUAST. cDNA encoding hPKD3 was shuttled as *Sac *I (blunted by T4 DNA polymerase)/*Not *I fragment into *Eco *RI (blunted)/*Not *I pUAST vector. All constructs were sequence verified. Several transgenic fly lines were generated for each construct and tested for their expression *in vivo*. Representative lines that had similar expression levels in imaginal discs as well as in Western blots were used for further experiments: PKD-WT^10-1^, PKD-kd^1–2^, PKD-kd^101-2^, PKD-SE^112-6^, PKD-SE^139-2^, dsPKD^114-5^, dsPKD^124-4^, hPKD2^II-2^, hPKD2^X-2^, hPKD3^I-3^, and hPKD3X^-1^.

### Generation of RNAi construct and transgenic lines

Cloning of a PKD RNAi construct followed the strategy outlined in before [20]. Using the primer pair dsPKDUP 5'-GGA TTC AAA CAG GAG GCG CAG CTG AAG AAC G-3' and dsPKDLO 5'-GGT ACC GGT GTT GGC GCT GCA GCT GAT TAA CTC-3', an 833 nucleotides (nt) spanning C-terminal part of the PKD cDNA (nt 1743 – 2576 starting with A_1_TG, corresponding to codons 582–836 plus 69 nt from 3' UTR) was cloned into pHIBS. It was shuttled in inverse orientation as *Bam *HI/*Kpn *I fragment into likewise opened pUAST vector after destroying an internal *Xho *I site within the 3' UTR of PKD (position 2537). In a second step, the PKD segment (nt 1743 – 2537) plus the Hairless intron [49] was cloned in direct orientation as *Sal *I/*Xho *I fragment into pUAST opened with *Xho *I. The final construct was sequence verified. Several transgenic lines were generated according to standard methods. Phenotypes were obtained at 25°C to 29°C in the presence of two or more copies of the dsPKD construct.

The DNA segment cloned in dsPKD showed an overall sequence identity of about 70% to both hPKD2 and hPKD3, respectively [13]. A pairwise alignment of hPKD2 with the *Drosophila *ds-segment showed a single 24 nucleotide stretch of full identity, however, with lower 5' than 3' stability of the sense strand, making it more likely guide than passenger strand. Moreover, we found 4 stretches of 19 or more nucleotides allowing a single mismatch. In contrast, the hPKD3-dsPKD pair showed a maximum of 11 identical nucleotides and not a single stretch of at least 19 identities allowing a single mismatch. Predictions for potent siRNAs were used to search for optimal pairs, however, not a even one was found that conforms to the signatures of a potent siRNA [41].

### Activity of PKD constructs in human tissue culture

#### Cell culture and transfection

HEK293T and COS7 cells were grown in RPMI supplemented with 10% fetal calf serum (FCS) in a humified atmosphere containing 5% CO_2_. HEK293T cells were transfected using TransIT293 reagent (Mirus) according to the manufacturer's instructions. For immunofluorescence, COS7 cells were grown on glass coverslips for 24 hours and transfected with Lipofectamine 2000 reagent (Invitrogen).

#### HRP transport assay

HEK293T cells were cotransfected with ss-HRP-Flag plasmid and empty pEGFP-N1 vector, pEGFP-N1-PKD-kd and pEGFP-N1-PKD-SE at a ratio of 1: 6.5, respectively. 24 h post-transfection cells were washed with serum-free media and HRP secretion was quantified after 0, 1, 3 and 6 h by incubation of clarified cell supernatant with ECL reagent (Pierce). Measurements were done with a luminometer (Lucy2, Anthos) at 450 nm.

#### Fluorescence microscopy

Cells were washed with phosphate buffered saline (PBS), fixed in 4% paraformaldehyde in PBS at room temperature for 10 min and washed with PBS. Coverslips were mounted in Fluoromount G (Southern Biotechnology) and cells were analyzed on a confocal laser scanning microscope (TCS SL, Leica) using 488 nm excitation and a 40.0/1.25 HCX PL APO objective lens. Images were processed with Adobe Photoshop.

### Expression analyses in fly tissues

PKD UAS-lines were induced ubiquitously using the da-Gal4 driver. Expression levels were monitored in Western blots loaded with protein extracts of each 100 first instar larvae using anti-GFP antibodies (Santa Cruz Biotechnology). For loading control, the blot was probed simultaneously with anti-gro antibodies (developed by C. Delidakis, obtained from DSHB, University of Iowa, Dept. Biol. Sci., Iowa City, IA 52242, USA). For *in situ *detection, PKD UAS-lines were crossed with *en-Gal4*, which drives expression in posterior compartments of imaginal discs. Discs were fixed for 5–10 minutes in 4% paraformaldehyde in phosphate buffered saline (PBS) and mounted in vectashield (Vector lab). To monitor downregulation of PKD by RNAi, the UAS-dsPKD^6-4 ^line was crossed with *ptc-Gal4 *and imaginal discs of third instar larval offspring processed according to standard methods [20] using mouse polyclonal anti-PKD antiserum [13]. The posterior compartment of wing discs was stained with monoclonal anti-En/Inv antibodies (Clone 4D9; developed by C. Goodman, obtained from DSHB, University of Iowa, Dept. Biol. Sci., Iowa City, IA 52242, USA). Apoptotic cells were labelled with anti-activated Caspase 3 antibodies (Cell Signaling Technology). Mitotic nuclei were detected with rabbit polyclonal antiserum directed against phosphorylated Histon H3 (Upstate Chemicals). BrdU incorporation was performed as described before [50] using monoclonal anti-BrdU antibodies (Clone G3G4, developed by S.J. Kaufman, obtained from DSHB). Developing photoreceptor cells were stained in eye imaginal discs using anti-Elav antibodies (Clone 7E8A10, developed by G. Rubin, obtained from DSHB). Secondary antibodies coupled to alkaline phosphatase, fluorescein or Cy3 were purchased from Jackson Laboratories (Dianova). Fluorescent tissue was analyzed by confocal microscopy (BioRad MRC1024 on Zeiss Axioskop). Figures were assembled using Corel Photo Paint and Corel Draw software.

### Phenotypic analyses

Samples of at least fifty flies of each genotype were inspected with low magnification microscopy (up to 40 fold). For closer examination, wings of ten or more flies were mounted and ten or more eyes were sectioned, respectively. Wings were dried in ethanol, mounted in Euparal (Merck), cleared over night and viewed with a Zeiss Axioskop using Normarski optics or phase contrast. Wing size, cell size and number were determined as described earlier [51], except that ImageJ software was used for pixel measurements. Fly heads were viewed with a WILD stereomicroscope. Pictures were taken with a Pixera digital camera (Optronics) using the Pixera Viewfinder Version 2.0 software. Sections of adult heads were performed as described before [52]. Figures were compiled using Corel Photo Paint and Corel Draw software.

## Authors' contributions

DM participated in the design of the study and experiments, carried out RNAi experiments and generated transgenic lines, assayed their expressivity, analyzed and interpreted the data and helped to draft the manuscript. ACN generated transgenic lines, planned, performed and interpreted immunoassays, growth and RNAi experiments and revised the manuscript. HG carried out overexpression and rescue experiments. AH designed and cloned the overexpression constructs, performed the secretion assay, provided mouse polyclonal PKD-antiserum and revised the manuscript. SJK carried out expression studies and growth experiments. IW carried out the light-dark experiments and prepared eye sections. AP conceived of the study, designed experiments, analyzed and interpreted the data and drafted the manuscript. All authors read and approved the final manuscript.
